# Practical Manual of Obstetrics

**Published:** 1884-08

**Authors:** 


### BOOK REVIEWS.

PRACTICAL MANUAL OF OBSTETRICS. *By* DR. E. VERRIER, *Lecturer on Obstetrics in the Faculty of Medicine of Paris. First American Edition, from the Fourth French Edition, with Revision and Annotations by* EDWARD L. PARTRIDGE, M. D., *Professor of Obstetrics in the New York Post-Graduate Medical School. April Number of Wood's Library; 8 vo., pp. xxi, 395. New York:* WILLIAM WOOD & CO. *1884.*

A *handbook* upon the art of midwifery is of questionable value, even when it is a methodical and exact *resume* of the principles and facts of the subject. When, however,—as in the present case,—the facts and principles of the art of midwifery are discussed without critical attention either to method or accuracy, its *raison d'etre* is clearly indemonstrable.

In the limited space at our command, we desire merely to allude to a few statements liable to criticism.

In the article upon the pelvic articulations, p. 6, the sacro-iliac and pubic articulations are termed symphyses, and no reference to synovial membrane is made in connection with the description of these joints. Luschka claims that these articulations are true joints, since the bones entering into their formation are expanded, covered by cartilage, held together by strong bands, or capsules, and the joint cavity is lined by synovial membrane. This view is sustained by the researches of Strauss and Muller of Vienna, in the demonstration of the presence of true joints in the same articulations among the domestic animals, in whose parturitions they are of great importance.

The dimensions of the false pelvis, as given on page 9, do not agree with the measurements of Carl Braun or Charpentier. On page 13, the angle of the female pubic arch is said to be 76°.

On page 99, the assertion is made that in first positions of face presentations,—that is, in right mento-posterior positions,—"the heart sounds are heard, as in the first position of the vertex, at the left of the abdomen, where (Depaul) "the back of the fœtus transmits the heart-beats to the ear of the listener."

Modern obstetrical authorities support the doctrine of Carl Braun: [1] "The fœtal heart-tones are always heard on the sternal surface of the fœtus, and on that side of the maternal abdomen toward which the point of the chin is directed."

On page 136, we are warned against Crede's method of placental delivery, except when "the cord is ruptured, or is very delicate and friable, or again, when we suspect an abnormal insertion."

"In the fever of recently delivered women," we are told, on page 141, that the patient must not "have too much fresh air."

The uncontrollable vomiting of pregnancy is stated, on page 151, as an indication for the induction of premature labor.

Bleeding is recommended on page 156, in eclampsia; on page 170, in abortion; on page 171, in hemorrhage; on page 172, to check uterine contractions; on page 283, in version.

The portion of the book devoted to operative obstetrics is incomplete in its discussion of conditions and indications for operative procedure.

Professor Pajot's preface and tables constitute the valuable

1. Carl Braun's Lehrbuch, d. g. Gynækologie, p. 188.

portions of the book, and will be received by the medical public with great satisfaction. Dr. Leigh H. Hunt, the translator, has discharged his duty in a faithful and felicitous manner.

The notes of the American editor present, as a rule, a pleasant contrast to the text. We do not agree with him, however, in his advice on page 235, to convert the face into a vertex presentation. However successful such efforts have been in the editor's hands, the practice is emphatically condemned by the great obstetrical authorities of the day.

Typographical errors are numerous and inexcusable.

Finally, if judgment must be passed, we do not think the book entitled to high consideration as a fair exposition of the subject, viewed absolutely or in relation to obstetrical doctrines in France. W. W. J.

